# The worldwide airline network and the dispersal of exotic species: 2007–2010

**DOI:** 10.1111/j.1600-0587.2008.05588.x

**Published:** 2009-02

**Authors:** Andrew J Tatem

**Affiliations:** Spatial Ecology and Epidemiology Group, Dept of Zoology, Univ. of OxfordSouth Parks Road, Oxford, OX1 3PS, UK, and Malaria Public Health and Epidemiology Group, Centre for Geographic Medicine, KEMRI/Wellcome Trust Research LaboratoriesP.O. Box 43640, 00100 Nairobi, Kenya

## Abstract

International air travel has played a significant role in driving recent increases in the rates of biological invasion and spread of infectious diseases. By providing high speed, busy transport links between spatially distant, but climatically similar regions of the world, the worldwide airline network (WAN) increases the risks of deliberate or accidental movements and establishment of climatically sensitive exotic organisms. With traffic levels continuing to rise and climates changing regionally, these risks will vary, both seasonally and year-by-year. Here, detailed estimates of air traffic trends and climate changes for the period 2007–2010 are used to examine the likely directions and magnitudes of changes in climatically sensitive organism invasion risk across the WAN. Analysis of over 144 million flights from 2007–2010 shows that by 2010, the WAN is likely to change little overall in terms of connecting regions with similar climates, but anticipated increases in traffic and local variations in climatic changes should increase the risks of exotic species movement on the WAN and establishment in new areas. These overall shifts mask spatially and temporally heterogenous changes across the WAN, where, for example, traffic increases and climatic convergence by July 2010 between parts of China and northern Europe and North America raise the likelihood of exotic species invasions, whereas anticipated climatic shifts may actually reduce invasion risks into much of eastern Europe.

Increasing rates of biological invasion are causing adverse economic, ecological and health effects globally (Lockwood et al. 2007). The mitigation of such effects requires an understanding of how humans facilitate the transport and establishment of exotic organisms (Floerl and Inglis 2005, McNeely 2006). International transport networks and hubs are especially important in providing movement routes and gateways into new regions (Drake and Lodge 2004, Tatem et al. 2006a, Tatem et al. 2006c). The establishment of a new route, as well as how often and how many individuals are transported on the route (“propagule pressure”), represent significant correlates of invasion success (Levine and D'Antonio 2003, Drake & Lodge 2004, Lockwood et al. 2005). Moreover, in connecting distant regions with differing biogeographic histories but similar climates, exotic organisms carried on such routes on international transport networks are more likely to survive upon arrival.

Recent increases in the rates of biological invasion and spread of infectious diseases have been linked to the continued expansion of the worldwide air transport network (WAN) (Liebhold et al. 2006, McCullough et al. 2006, Tatem et al. 2006c, Lines 2007), with pest species interception rates related to incoming air traffic volumes (Liebhold et al. 2006). Among others, the Mediterranean fruit fly *Ceratitis capitata* has been consistently imported in airline baggage (Liebhold et al. 2006), plant pathogens are often found in air cargo (McCullough et al. 2006) and disease carrying mosquitoes have survived long haul flights in aircraft cabins (Lounibos 2002; Tatem et al. 2006b). Historically, natural barriers and distance restricted the long distance movement of organisms, but the WAN has reduced such effects, leaving traffic and climate as principal constraints to the spread of those organisms sensitive to the weather. Both of these constraints, however, are undergoing change and will continue to do so in the near future. International passenger numbers are anticipated to rise by an annual average rate of 4.8% (IATA 2006b), increasing propagule pressure. At the same time, global temperatures are predicted to rise by around 0.2°C per decade with consequent effects on rainfall and humidity levels (IPCC 2007). Moreover, recent studies have shown past interception rates to be inversely related to the gross national income (GNI) of origin countries (Liebhold et al. 2006). Such variations and changes, combined with the limited funds available for surveillance and control, highlight the need for both an understanding of the complex spatiotemporal dynamics of traffic flows and climate linkages on the WAN, and multidisciplinary approaches that can extract relevant information on future changes in biological invasion risks on specific routes.

Analyses of the present day structure of the WAN in terms of traffic volumes and the way regions with similar climates are connected has shown there to be great intra-annual variation (Tatem and Hay 2007). Climatic similarity across the WAN is skewed (most geographically close airports are climatically similar) but heavy-tailed (there are considerable numbers of routes connecting geographically distant airports with similar climates), with overall climatic similarity highest in the June–August period, matching the annual peak in air traffic. Climatically matched, geographically distant airports form sub-networks within the WAN that change throughout the year. Further, accounting for passenger and freight traffic data highlights as at greater risk of invasion those airports that are climatically well connected by numerous high capacity routes. Here, these analyses are extended to examine the magnitude and direction of anticipated changes between today and 2010.

The spatial and seasonal distribution of incoming traffic volumes to individual airports and how these are expected to change by 2010 are explored. Combining these data with climate scenarios enables the identification of scheduled routes within the WAN that are anticipated to both carry more traffic and link regions that are becoming more similar climatically (potential invasion “risk routes”). Further, the seasonal mapping of those airports with multiple incoming “risk routes” provides an indication of the direction and magnitude of regional invasion risk changes. Finally, the interrelation between changing traffic levels and the GNIs of the countries of origin are examined briefly as a further indicator of spatially varying potential invasion risks.

## Materials and methods

### WAN data

Flight schedule data from the 800+ airlines of the world for the 12 month period of 1 June, 2006 to 30 May, 2007 were obtained from the OAGMAX database <http://www.oagdata.com/solutions/max.aspx>. This database is compiled by OAG Worldwide (Downers Grove, IL), and includes all scheduled flights and scheduled charter flights, both for large (air carriers) and small aircraft (air taxis). Stopover airports on scheduled routes are not included in the database. During the period considered, there were 35 142 541 scheduled flights operating between 3570 airports on over 44 000 different routes.

Passenger and freight forecast data for the period 2006–2010 were obtained from the International Air Transport Association (IATA) (IATA 2006a,b). These included predicted country to country percentage changes in passenger numbers and freight tonnage for each year. For the seat capacities of each individual route carrying passengers in the OAGMAX dataset (97.1% of all flights), the percentage changes in passenger numbers were applied, based on the origin and destination countries, to obtain monthly seat capacity estimates for 2007, 2008, 2009 and 2010. For the remaining 2.9% of flights that carried only freight, the predicted freight tonnage percentage changes were applied to the total tonnage measures to obtain monthly tonnage estimates for 2007, 2008, 2009 and 2010. The strong relationship between tonnage and seat capacity (Tatem and Hay 2007) was then used to convert these estimates to equivalent and comparable seat capacities to ensure their inclusion in the analyses. Though it is anticipated that new routes will be developed, projections of these are not available, and traffic growth on existing routes will likely prove a far more significant aspect of WAN growth. Given the structures and laws currently in place, it is also likely that the 2010 WAN will exhibit a very similar architecture to that of today's WAN.

### Climatic distances

Gridded monthly climate scenario data from the HadCM3 coupled atmosphere–ocean general circulation model (AOGCM) developed at the Hadley Centre for Climate Prediction and Research were obtained from <www.mad.zmaw.de/IPCC_DDC/html/SRES_TAR/index.html>. With this study only examining changes up until 2010, the use of data from just a single model has little consequence on results, as the difference between the projected impacts of each model are tiny on this timescale (IPCC 2007). The medium–high A2 emission scenario was used, which represents a continuation of modern day economic development with increasing world population. This choice of scenario also has little consequence on results as the difference between the projected impacts of emission scenarios before 2030 are tiny (Stott and Kettleborough 2002, IPCC 2007). Since the HadCM3 AOGCM simulates weather, individual years can be noisy. Therefore, 11-yr averages centred on 2007 (2002–2012) for the baseline, and 2008 (2003–2013 inclusive), 2009 (2004–2014 inclusive) and 2010 (2005–2015 inclusive) for future climate, were used. Moreover, the average of the A2a, A2b and A2c ensemble member runs, each of which are runs of the A2 emission scenario initialised with very small differences in their starting conditions, were used to obtain a more representative climatology. The specific meteorological variables used were mean surface air temperature (K) at 2 m, total precipitation (mm d^−1^) and relative humidity (%) for the years 2002–2015 inclusive. These three variables provide a basic representation of the climatic regime of an area (Rogers and Randolph 2003), with pest species sensitive to small changes in all three. No upscaling of the 2.5°×3.75° (latitude by longitude) spatial resolution of the data was undertaken, given that the aim of the study was to examine regional changes and that national-level passenger and freight forecasts were used.

The climate scenario surfaces were linearly rescaled to the common data range, 0–1, and the locations of the airports were superimposed onto them. Within the climate surfaces, the grid square covering the location of each airport was identified. Given the climate grid used, some mainland airports were shifted to their nearest grid square.

Any airports located on islands too small to be represented by the climate surfaces were eliminated from the analysis, reducing the sample size to 2635 airports. The remaining network included 99.96% of the global seat capacity from the original database. For each month, the selected grid square data from the climate surfaces thus formed the climatic “signature” of each airport.

The climatic similarity of a scheduled air route connecting airport *i* with airport *j* in the WAN is defined here initially by the Euclidean distance between the climatic conditions at *i* and those at *j*. A relatively high climatic Euclidean distance (CED) on a route shows that the regions connected are climatically dissimilar for the month in question, while a relatively low CED indicates climatic similarity. The Euclidean distance in climatic space between each airport signature and every other airport signature was calculated to produce 48 symmetrical airport “climatic dissimilarity” matrices, one for each month of each year (2007–2010), with each cell representing a CED between one airport and another. Euclidean distance measures were used because the size of the climate scenario grid squares meant just one grid square per airport and hence, too few data values were available for any more sophisticated measures of environmental distance. While such individual monthly CEDs provided an indication of the chances of survival of a climatically-sensitive exotic organism upon arrival, they do not indicate the potential for continued survival and establishment given climatic conditions for the remainder of the year. Thus, annual climatic similarity between origin and destination locations was estimated by calculating the per-route mean annual CED and scaling each monthly CED by it. This had the effect of substantially increasing monthly CEDs for routes connecting regions that are climatically dissimilar over the course of a year. For instance, considering just month-specific CED, the Paris–Abidjan and Paris-Beijing routes both exhibit similar, relatively low CEDs for August. However, after scaling these CEDs by mean annual CED, the Paris–Beijing August CED is significantly lower that that of the Paris–Abidjan route, reflecting more realistically the monthly and annual climatic similarity between the regions, and the chances of exotic organism survival. Finally, these scaled CEDs between airports where no scheduled route exists in the WAN were removed from the dissimilarity matrices.

### Climatic similarity and traffic indices

Tatem and Hay (2007) proposed a set of indices for the quantification and combination of features of the WAN proven important to its role in biological invasion. Brief details of each index are provided here along with modifications made for these analyses, but readers are referred to the original paper for full descriptions.

To examine the general, combined effect of climatic similarity and traffic volume, for each route and month, traffic-scaled climatic Euclidean distance, CEDt, was calculated. For each month, the traffic-scaled climatic Euclidean distance between airport *i* and *j*,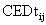, is defined as:

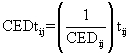


where 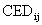 is the climatic Euclidean distance between airport *i* and *j*, scaled by annual CED as described above, and 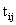 is the total monthly seat capacity on the route from airport *i* to airport *j*.

While CEDt identifies potential high risk routes within the WAN for dispersal and establishment of climatically sensitive biota, those airports with multiple scheduled links to climatically similar regions were also identified to highlight airports with elevated invasion risks. For each month, the “climatic similarity Index” (CSI) of an airport *i* is defined as:

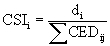


where,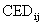 is the climatic Euclidean distance between airport *i* and *j*, scaled by annual mean CED. The degree 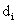
of airport *i* within the WAN is defined as the number of other airports to which it is connected by incoming direct scheduled routes.

The traffic-scaled climatic similarity index of airport *i*,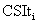, provides a relative indicator of those airports with high incoming traffic routes from regions with similar climate to its own. It is defined as:

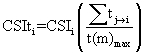


where 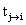 is the total monthly seat capacity incoming to airport *i* from airport *j*, and 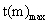 is the global maximum total seat capacity for month m.

### Origin GNI analysis

Liebhold et al. (2006) found pest species interception rates to be inversely related to GNI. While data on interceptions are likely to be biased, making quantitative links to exotic species importation events difficult, the current and future interrelations between GNIs, the WAN architecture and traffic levels are briefly explored here for illustrative purposes. GNI data for every country of the world were obtained from the World Bank development indicators database (World Bank 2008). For each route in the scheduled flight database, the GNI of the country of origin was identified. This GNI was divided by the seat capacity to create monthly per-seat GNIs for each route. Finally, for every airport, the per-seat GNIs of every incoming route were summed and then divided by the number of incoming routes to provide an average per-seat incoming GNI.

## Results

### Large-scale WAN changes 2007–2010

**Figure 1:**
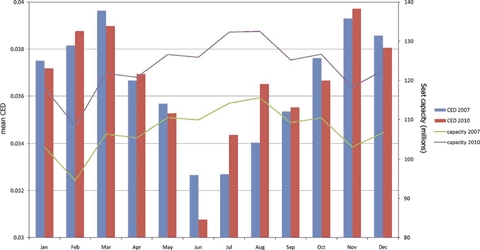
Mean monthly CEDs and monthly total seat capacities across the WAN for 2007 and 2010.

Figure 1 demonstrates the large increases in traffic expected on the WAN over the next 3 yr. Average monthly percentage change in seat capacity from 2007 to 2010 is +14.74%, while the equivalent average change in CED is just +0.74%, equating to an overall drop in climatic similarity. The plot shows that July and August are expected to remain the months of peak traffic in 2010 and consequently, propagule pressure, with global seat capacity between 2007 and 2010 increasing the most in July (+15.87%) and peaking at over 132 million in August 2010. In contrast, predicted changes in global climate are relatively small, but show month-to-month variations. As previously described (2007), June–August is the period of overall highest climatic similarity (i.e. lowest CEDs) at present. However, anticipated climatic changes mean that by 2010, June is likely to be the month of overall lowest CEDs, while the WAN in July and August will on average connect regions of more dissimilar climate than in 2007. This suggests that climatically sensitive organisms moving on the WAN in 2010 will be, overall, more likely to find their arrival destinations habitable in June.

### Incoming traffic changes 2007–2010

While Fig. 1 demonstrates that overall levels of traffic on the WAN are expected to increase substantially between 2007 and 2010, Fig. 2 shows that large regional variations in incoming traffic volumes exist. The greatest increases in incoming traffic are expected to be seen at airports across China, India, Russia and eastern Europe, where numbers are likely to increase by over 25%, providing many more opportunities for the importation of exotic species. Large rises are also expected for airports in Mexico, north Africa and southeast Asia, whereas today's busiest airports in the US and western Europe are expected to show average increases in incoming seat capacity of<15% over the 2007–2010 period.

**Figure 2:**
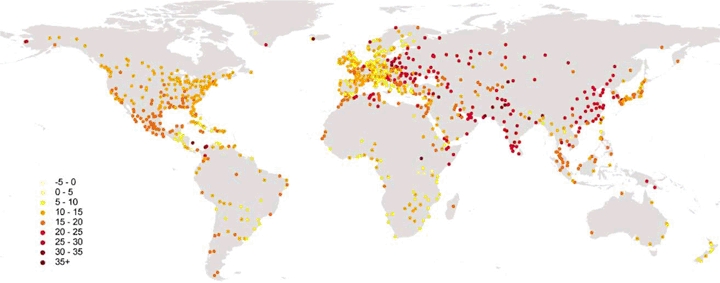
Percentage change in incoming seat capacities 2007–2010 at airports globally.

### Traffic-scaled climatic Euclidean distance changes 2007–2010

Flight routes that link spatially distant but climatically similar regions can promote biological exchange between areas where separation has resulted in different species assemblages adapted to the same climate. High traffic volumes on these routes increase propagule pressure and increase the risks of biological invasion further. Those routes of greatest flight distance and highest CEDt were therefore identified. For the top 10% of routes by greatest distance, Table 1 shows those routes with the highest CEDt values for January, April, July and October in 2007 and 2010, and those routes where CEDt shows the greatest increases over the period. As highlighted in Tatem and Hay (2007), each month shows very different sets of routes as seasonal cycles mean changes in those regions most similar climatically to each other. Substantial changes in those routes with the largest CEDt values between 2007 and 2010 are also seen however. The majority of these changes reflect the anticipated large rises in traffic and consequently, risk of exotic species transportation, on routes to and from east Asia, particularly China (Fig. 2), but also anticipated climatic convergence between regions, increasing biological invasion risks.

**Table 1:** From the top 10% of routes by longest distance: (a) top 10 routes by CEDt for January, April, July and October 2007, (b) top 10 routes by CEDt for January, April, July and October 2010, (c) top 10 routes by largest difference between CEDt in 2007 and 2010 for January, April, July and October.

Rank	January	April	July	October
(a)				
1	Los Angeles (USA) – Shanghai (China)	Portland (USA) – Frankfurt (Germany)	Montreal (Canada) – Paris (France)	Detroit (USA) – Osaka (Japan)
2	New York (USA) – Milan (Italy)	Seattle (USA) – Frankfurt (Germany)	Toronto (Canada) – Frankfurt (Germany)	Detroit (USA) – Nagoya (Japan)
3	Seattle (USA) – Frankfurt (Germany)	Seattle (USA) – Copenhagen (Denmark)	Montreal (Canada) – Munich (Germany)	Chicago (USA) – Tokyo (Japan)
4	New York (USA) – Zurich (Switzerland)	Seattle (USA) – Paris (France)	New York (USA) – Vienna (Austria)	Minneapolis (USA) – Tokyo (Japan)
5	Los Angeles (USA) – Amsterdam (Netherlands)	Seattle (USA) – London (UK)	Montreal (Canada) – London (UK)	Boston (USA) – Nagoya (Japan)
6	New York (USA) – Hamburg (Germany)	Johannesburg (S Africa) – Sydney (Australia)	Toronto (Canada) – Paris (France)	Boston (USA) – Tokyo (Japan)
7	Istanbul (Turkey) – Shanghai (China)	Chicago (USA) – Frankfurt (Germany)	Ottawa (Canada) – London (UK)	New York (USA) – Tokyo (Japan)
8	Los Angeles (USA) – Taipei (Taiwan)	Washington DC (USA) – Tokyo (Japan)	Frankfurt (Germany) – Beijing (China)	Amsterdam (Netherlands) – Osaka (Japan)
9	New York (USA) – Geneva (Switzerland)	Portland (USA) – London (UK)	New York (USA) – Brussels (Belgium)	Houston (USA) – Delhi (India)
10	Toronto (Canada) – Zurich (Switzerland)	New York (USA) – Milan (Italy)	Toronto (Canada) – Dusseldorf (Germany)	Washington DC (USA) – Tokyo (Japan)
(b)				
1	Los Angeles (USA) – Amsterdam (Netherlands)	Seattle (USA) – London (UK)	Montreal (Canada) – Munich (Germany)	Seattle (USA) – Seoul (Korea)
2	New York (USA) – Milan (Italy)	Portland (USA) – London (UK)	Montreal (Canada) – Paris (France)	Detroit (USA) – Osaka (Japan)
3	Istanbul (Turkey) – Shanghai (China)	Seattle (USA) – Copenhagen (Denmark)	Toronto (Canada) – Beijing (China)	Minneapolis (USA) – Tokyo (Japan)
4	Seattle (USA) – Frankfurt (Germany)	Portland (USA) – Frankfurt (Germany)	New York (USA) – Vienna (Austria)	Detroit (USA) – Nagoya (Japan)
5	Los Angeles (USA) – Shanghai (China)	Washington (USA) – Tokyo (Japan)	New York (USA) – Beijing (China)	Chicago (USA) – Tokyo (Japan)
6	New York (USA) – Zurich (Switzerland)	Seattle (USA) – Frankfurt (Germany)	Frankfurt (Germany) – Beijing (China)	Boston (USA) – Nagoya (Japan)
7	New York (USA) – Hamburg (Germany)	New York (USA) – Seoul (Korea)	Chicago (USA) – Beijing (China)	Boston (USA) – Tokyo (Japan)
8	New York (USA) – London (UK)	Chicago (USA) – Frankfurt (Germany)	Toronto (Canada) – Frankfurt (Germany)	New York (USA) – Tokyo (Japan)
9	Boston (USA) – Paris (France)	New York (USA) – Milan (Italy)	Montreal (Canada) – Prague (Czech Rep)	Amsterdam (Netherlands) – Osaka (Japan)
10	Chicago (USA) – Warsaw (Poland)	Chicago (USA) – Paris (France)	Chicago (USA) – Warsaw (Poland)	San Francisco (USA) – Shanghai (China)
(c)				
1	Los Angeles (USA) – Amsterdam (Netherlands)	Seattle (USA) – London (UK)	Montreal (Canada) – Munich (Germany)	Seattle (USA) – Seoul (Korea)
2	Istanbul (Turkey) – Shanghai (China)	New York (USA) – Seoul (Korea)	Toronto (Canada) – Beijing (China)	San Francisco (USA) – Shanghai (China)
3	Las Vegas (USA) – London (UK)	Portland (USA) – London (UK)	Toronto (Canada) – Seoul (Korea)	Minneapolis (USA) – Tokyo (Japan)
4	Rome (Italy) – Shanghai (China)	Chicago (USA) – Seoul (Korea)	New York (USA) – Beijing (China)	Atlanta (USA) – Rome (Italy)
5	Boston (USA) – Milan (Italy)	Los Angeles (USA) – Beijing (China)	Vancouver (Canada) – Amsterdam (Netherlands)	Detroit (USA) – Shanghai (China)
6	New York (USA) – Frankfurt (Germany)	London (UK) – Minneapolis (USA)	Munich (Germany) – Beijing (China)	Detroit (USA) – Osaka (Japan)
7	Chicago (USA) – Warsaw (Poland)	Atlanta (USA) – Singapore (Singapore)	Detroit (USA) – Beijing (China)	Philadelphia (USA) – Hong Kong (China)
8	Toronto (Canada) – Milan (Italy)	Detroit (USA) – Shanghai (China)	Chicago (USA) – Beijing (China)	New York (USA) – Seoul (Korea)
9	Salt Lake City (USA) – Milan (Italy)	Chicago (USA) – Warsaw (Poland)	Toronto (Canada) – Munich (Germany)	Istanbul (Turkey) – Shanghai (China)
10	New York (USA) – Prague (Czech Rep)	Munich (Germany) – Shanghai (China)	Chicago (USA) – Warsaw (Poland)	Chicago (USA) – Shanghai (China)

The largest CEDt values for January in both 2007 and 2010 are exhibited by the high traffic routes between climatically similar regions of the US and Europe. Table 1c demonstrates, however, that some of the greatest increases in January CEDt values between 2007 and 2010 are on routes connecting regions where climatic similarity remains high, but where traffic levels are expected to grow fastest, including China, the Czech Republic and Poland. Similar results are seen for April, with for example, South Korea's climatic similarity to northeast USA and large traffic increases on routes between the two countries causing the Seoul to New York and Chicago routes to appear in Table 1(c).

Climatic similarity between the most populous parts of China and northern Europe or eastern North America is highest in July (Tatem and Hay 2007). Whilst traffic volumes on routes linking the regions are presently not as high as many North America to Europe routes, increases in air traffic to and from China mean that by 2010, four of the top 10 long distance routes by July CEDt involve Beijing (Table 1b), indicating an increasing risk of exotic organism exchange between the regions. Finally, October's top 10 routes in Table 1a, b are dominated by routes that connect eastern USA with Japan, where traffic levels and climatic similarity in October remain high. Of note however are those routes showing the largest increases in CEDt between 2007 and 2010 for October. Again, routes connecting the US with China feature, and with a 21% increase in traffic and 77% increase in CED due to predicted precipitation and humidity changes, it is the Seattle to Seoul route that has the largest CEDt values in 2010 and, consequently, increasing risks of exotic species moving between the regions and establishing upon arrival.

### Traffic-scaled climatic similarity index changes 2007–2010

**Figure 3:**
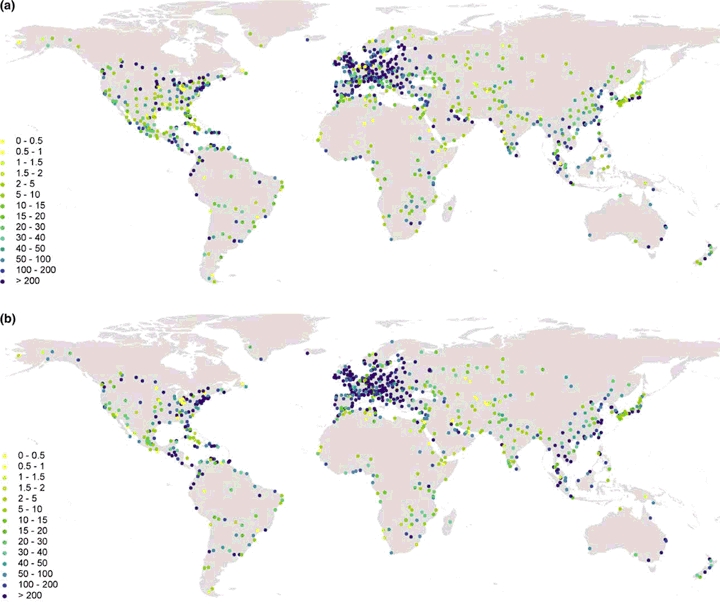
CSIt in 2007 for (a) January and (b) July. Percentage change in CSIt 2007–2010 for (c) January and (d) July. Results for April and October are presented in Supplementary Information.

Figure 3 shows how January and July CSIt values for each airport are anticipated to change by 2010. Month-specific CSIt values for 2007 (Fig. 3a, b) are highest at those airports where there are numerous incoming high traffic routes from regions with similar climatic regimes to the airport in question, combining the dual aspects of propagule pressure and climatic suitability in producing an indication of invasion risk. Accounting for the regionally varying climatic and incoming traffic changes expected by 2010, Fig. 3c, d show that some substantial shifts in CSIt are likely to occur.

The overwhelming majority of airports show CSIt increases between 2007 and 2010, as incoming traffic volumes increase and, consequently, risk of exotic species importation rises. However, Fig. 3c shows that for January, many airports in north-central USA, central Mexico, east and southeast Africa, Italy and southeast Europe show drops in CSIt from 2007 to 2010. In contrast, the CSIt of airports across northern India, Malaysia and Indonesia all increase by over 50%. July is anticipated to see more substantial changes than January, with CSIt in July 2010 dropping by over 30% from July 2007 values for certain airports in northeastern USA, southern Africa and eastern Europe in particular, as their regional climates diverge from those that each are connected to by high traffic routes. Nevertheless, anticipated traffic volume increases and climatic changes contribute to >50% rises in July CSIt at west coast North American and many east Asian airports (Fig. 3d), potentially resulting in large increases in the risks of arrival and establishment of exotic species in these regions.

### Changes in incoming GNI 2007–2010

**Figure 4:**
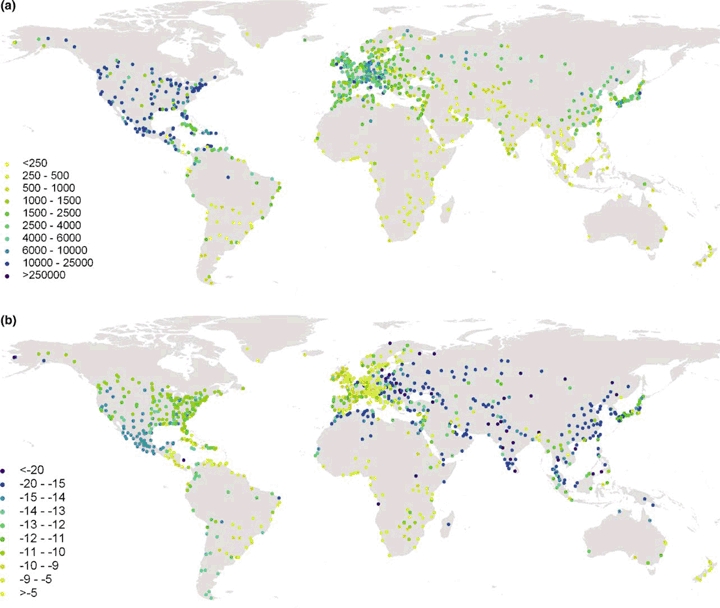
(a) Average per-seat incoming gross national income (GNI) in US$ for 2007, (b) percentage change in incoming average per-seat GNI 2007–2010.

Figure 4a shows, unsurprisingly, that those regions of the world with the lowest GNIs also contain the airports with the lowest average incoming per-seat GNIs. Nevertheless, some note-worthy spatial outliers exist, especially the lower incoming per-seat GNIs at the major international hub airports of North America and Europe that have high traffic links to South America and Africa, respectively. While relationships found between GNI and invasion risk do remain unvalidated and subject to bias, evidence does suggest that the lower average incoming GNIs make them at higher risk of exotic species importation. It is also these hubs that are likely to see the biggest drops in average incoming GNI per seat by 2010, as anticipated traffic rises from lower GNI countries occurs (Fig. 4b). These traffic increases (Fig. 2) also mean that lower GNI countries will become more interconnected, resulting in significant average incoming per-seat GNI reduction, and consequently, further potential for rises in invasion risks.

## Discussion

The WAN enables many of the world's most isolated and diverse ecosystems to become connected and aid the movement of organisms to new habitats where they can become damaging invasive species. The most feared of these species can cause damage worth billions of dollars (Pimental et al. 2000), compromise national biosecurity (Biosecurity Council 2003), erode native biological diversity (Gewin 2005) and affect human health (Tatem et al. 2006c). With the continued growth of air travel and rapid climatic changes, the threat from invasive species is likely to increase, and our best hopes for minimizing such damage remain through the optimization of prediction, surveillance and control methods at international transport gateways. Quantifying how, when and where heightened risks of biological invasion may occur in the future enables surveillance and control authorities to target limited resources. Here, the likely future directions and spatial distribution of changes in two of the most important aspects of biological invasion through the WAN, transportation risk and climatic similarity (Tatem et al. 2006a, Tatem and Hay 2007), have been examined for the first time. The former represents a surrogate for propagule pressure (Kolar and Lodge 2001, Lockwood et al. 2005), however, it should be noted that in certain regions, such as Australia and New Zealand, the increases in transportation risks have been mirrored by a rise in border management, which has effectively constrained propagule pressure.

The results outlined in this paper build upon those presented by Tatem and Hay (2007) by examining the combined effects of anticipated climate change and air traffic growth on monthly climatic similarity and biological exchange across the WAN. Moreover, overall annual climatic similarity is accounted for here, enabling refined measures of both the likelihood of organism survival upon arrival and potential for establishment based on climate. Results show that the regionally varying changes in traffic flows and climates connected are likely to have significant effects on the role of the WAN in future biological invasions. Overall, the architecture and anticipated traffic flows of the WAN mean that the dual-effects of peak traffic and lowest CED values in the Northern Hemisphere summer months, raising overall invasion risks, are maintained from 2007 to 2010 (Fig. 1). However, regional variations in predicted climate change result, on average, in WAN routes in July and August 2010 displaying higher CEDs than in 2007, while June 2010 routes link more similar regions climatically. Thus, climatically sensitive organisms travelling on the WAN in 2010 are, on average, more likely to find their destinations hospitable in June than any other month. The regional variations driving the decrease in average CED between July 2007 and July 2010 are mapped in Fig. 3d. Despite traffic increases (Fig. 2), many airports in eastern Europe and south-eastern Africa show decreases in CSIt, caused by climatic divergence from those regions each is connected to on the WAN. Nevertheless, it is clear that many of these decreases are balanced by substantial increases across east Asia and coastal North America, underlining the heterogeneity in changing invasion risks likely over the next three years.

Figure 2 shows that the rapidly growing economies of China, India, Russia, Mexico, eastern Europe, the Middle East and northern Africa are expected to provide the largest increases in air traffic. With growth of over 30% by 2010, organisms will have many more opportunities to move between these regions and those connected by air routes to them, translating into substantial overall increases in transportation risk and consequently, propagule pressure, driven by the WAN. Whether such organisms establish in new regions depends initially upon finding climatic conditions that match as closely as possible those at their origin. Table 1 and Fig. 3 suggest, though, that many air routes from these growing economies are providing links to climatically similar new regions, dependent upon the time of year. Table 1c in particular reveals that routes to and from China dominate those showing the largest increases in CEDt, corresponding to the greatest increases in climatically-sensitive organism invasion risk. The effects of rapid economic growth on increasing rates of biological invasion both within (Lin et al. 2007) and from (Normile 2004) China have already been observed. Results here suggest that these effects, through the WAN at least, are likely to increase further by 2010, with Chinese airports among ‘invasion hotspot’ airports (Fig. 3) and different routes for each season maintaining high CEDt links between China and parts of Europe and North America (Table 1).

The results presented in Fig. 4 illustrate an additional aspect to exotic species importation risk across the WAN, and this is an area towards which future research should be directed. Largescale surveys in the US have shown that people from different countries exhibit different behaviours that make them more or less likely to transport exotic organisms (Liebhold et al. 2006, McCullough et al. 2006). Though such surveys are based on interceptions and thus open to bias, inverse relationships between pest importation and GNI have been found, and Fig. 4 illustrates the potential additional spatial variation in invasion risks that this brings, given the architecture and traffic flows of the 2007 and 2010 WAN. Fig. 4 shows that the large hub airports in North America and Europe that provide high traffic links to South America, Africa and southeast Asia exhibit significantly lower average incoming per-seat GNIs than the surrounding airports that deal with more local traffic. Further research is required to ascertain whether, and how, these routes from lower GNI countries translate into higher transportation rates of exotic organisms, but the results suggest that these hub airports are at greater risk. Such risks are likely to increase as traffic from low GNI countries increases, and the traffic rises between low GNI countries also exacerbates potential invasion risks for such countries.

With incoming air traffic volumes related to pest species interception rates (Liebhold et al. 2006), the anticipated rises in traffic across the WAN over the next three years are likely to result in substantially increased propagule pressure and rates of exotic species introduction. Combined with expected changes in global climate, risks of biological invasion driven by the WAN will change rapidly. Our best hopes of minimizing such invasions and the environmental, economic and health impacts they can cause, are through improved surveillance, prediction and control at international transport gateways. The relative success of strong existing surveillance systems (Biosecurity Council 2003, Biosecurity Australia 2007) demonstrate how this can be achieved.

## Supplementary Material

Download the Supplementary material as file E5588 from<www.oikos.ekol.lu.se/appendix>.


