# Supplementary material for: The worldwide airline network and the dispersal of exotic species: 2007–2010
Source: Ecography. 2009 Feb;32(1):94–102. doi: 10.1111/j.1600-0587.2008.05588.x (PMC2836027; doi:10.1111/j.1600-0587.2008.05588.x)
Supplement: Supplementary file 1 [file eco0032-0094-appx.pdf]

Ecography

**E5588**
Tatem, A. J. 2009. The worldwide airline network and the dispersal of exotic species: 2007–2010. – Ecography 32: 94–102.

# Supplementary material

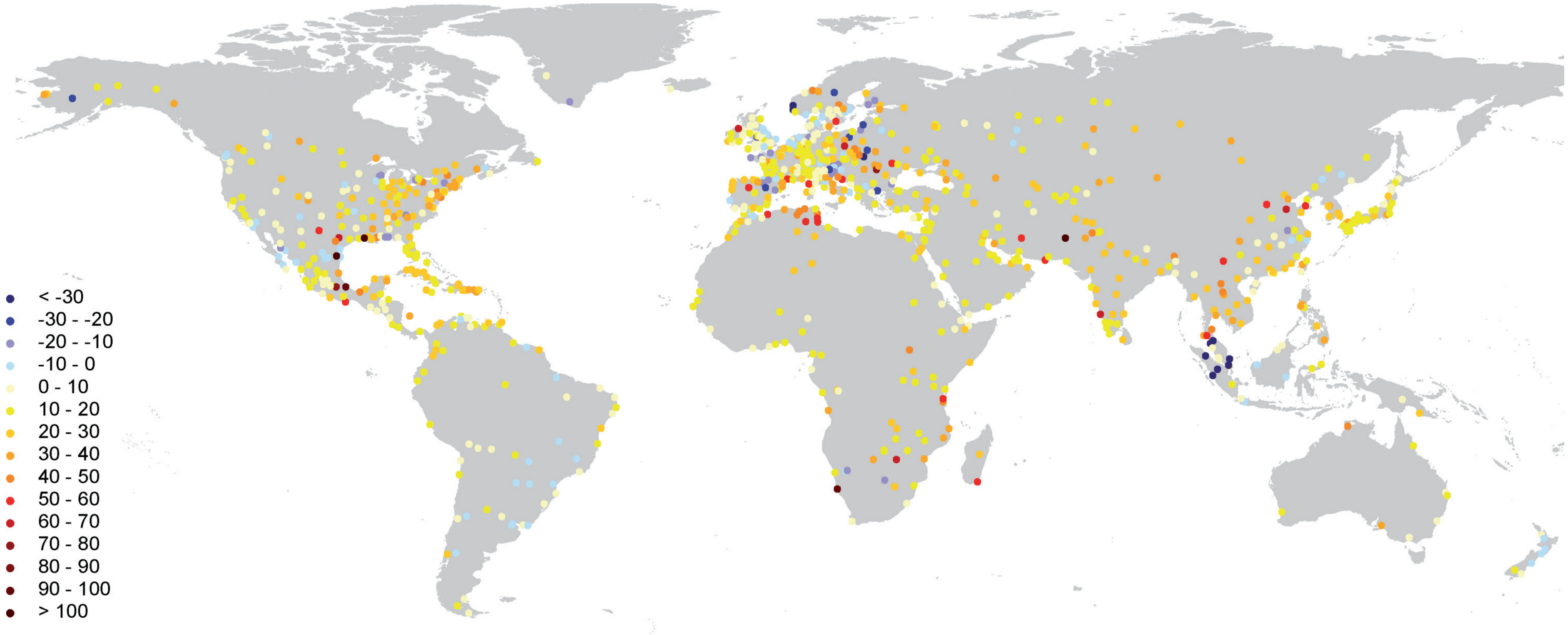


Figure S1. Percentage change in CSIt 2007–2010 for April.

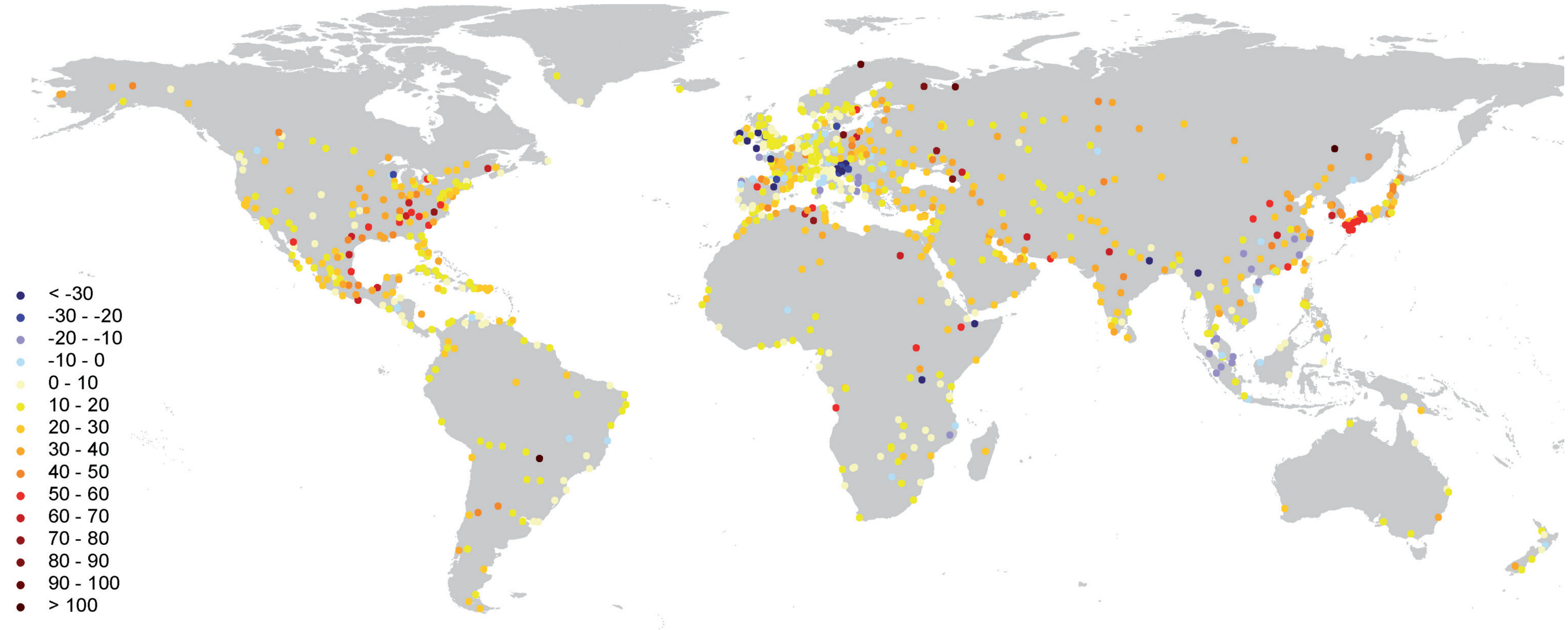


Figure S2. Percentage change in CSIt 2007–2010 for October.
